# Characterization of subclinical diastolic dysfunction by cardiac magnetic resonance feature-tracking in adult survivors of non-Hodgkin lymphoma treated with anthracyclines

**DOI:** 10.1186/s12872-021-01996-6

**Published:** 2021-04-12

**Authors:** Maurício Fregonesi Barbosa, Daniéliso Renato Fusco, Rafael Dezen Gaiolla, Konrad Werys, Suzana Erico Tanni, Rômulo Araújo Fernandes, Sergio Marrone Ribeiro, Gilberto Szarf

**Affiliations:** 1 Department of Diagnostic Imaging, Universidade Federal de São Paulo (UNIFESP), Rua Napoleão de Barros 800, Vila Clementino, São Paulo, 04024-002 Brazil; 2 Department of Tropical Diseases and Diagnostic Imaging, Universidade Estadual Paulista (UNESP), Botucatu, Brazil; 3 Cardiology Division, Internal Medicine Department, Universidade Estadual Paulista (UNESP), Botucatu, Brazil; 4 Hematology Division, Internal Medicine Department, Universidade Estadual Paulista (UNESP), Botucatu, Brazil; 5 University of Oxford Centre for Clinical Magnetic Resonance Research (OCMR), University of Oxford, Oxford, UK; 6 Pneumology Division, Internal Medicine Department, Universidade Estadual Paulista (UNESP), Botucatu, Brazil; 7 Department of Physical Education, Universidade Estadual Paulista (UNESP), Presidente Prudente, Brazil; 8 Hospital Israelita Albert Einstein, São Paulo, Brazil

**Keywords:** Strain, Feature-tracking, Diastolic dysfunction, Cancer, Chemotherapy

## Abstract

**Background:**

The use of anthracycline-based chemotherapy is associated with the development of heart failure, even years after the end of treatment. Early detection of cardiac dysfunction could identify a high-risk subset of survivors who would eventually benefit from early intervention. Cardiac magnetic resonance feature-tracking (CMR-FT) analysis offers a practical and rapid method to calculate systolic and diastolic strains from routinely acquired cine images. While early changes in systolic function have been described, less data are available about late effects of chemotherapy in diastolic parameters by CMR-FT. The main goal of this study was to determine whether left ventricular (LV) early diastolic strain rates (GDSR-E) by CMR-FT are impaired in long-term adult survivors of non-Hodgkin lymphoma (NHL). Our secondary objective was to analyze associations between GDSR-E with cumulative anthracycline dose, systolic function parameters and myocardial tissue characteristics.

**Methods:**

This is a single center cross-sectional observational study of asymptomatic patients in remission of NHL who previously received anthracycline therapy. All participants underwent their CMR examination on a 3.0-T scanner, including cines, T2 mapping, T1 mapping and late gadolinium enhancement imaging. Derived myocardial extracellular volume fraction was obtained from pre- and post-contrast T1 maps. CMR-FT analysis was performed using Trufi Strain software. The data obtained were compared between anthracycline group and volunteers without cardiovascular disease or neoplasia.

**Results:**

A total of 18 adult survivors of NHL, 14 (77.8%) males, at mean age of 57.6 (± 14.7) years-old, were studied 88.2 (± 52.1) months after exposure to anthracycline therapy (median 400 mg/m^2^). Compared with controls, anthracycline group showed impaired LV global early diastolic circumferential strain rate (GCSR-E) [53.5%/s ± 19.3 vs 72.2%/s ± 26.7, *p* = 0.022], early diastolic longitudinal strain rate (GLSR-E) [40.4%/s ± 13.0 vs 55.9%/s ± 17.8, *p* = 0.006] and early diastolic radial strain rate (GRSR-E) [− 114.4%/s ± 37.1 vs − 170.5%/s ± 48.0, *p* < 0.001]. Impaired LV GCSR-E, GLSR-E and GRSR-E correlated with increased anthracycline dose and decreased systolic function. There were no correlations between GDSR-E and myocardial tissue characteristics.

**Conclusions:**

Left ventricular early diastolic strain rates by CMR-FT are impaired late after anthracycline chemotherapy in adult survivors of non-Hodgkin lymphoma.

## Background

The use of anthracycline chemotherapy is associated with the development of heart failure (HF) among survivors of non-Hodgkin lymphoma (NHL), even years after the end of treatment [1]. Detecting cardiac dysfunction could identify a subgroup of asymptomatic patients at high risk for HF who would eventually benefit from early intervention [2–4]. The most common method of monitoring cardiac function during and after cancer treatment is the measurement of left ventricular (LV) ejection fraction (EF) by 2-dimensional transthoracic echocardiography (TTE) [5, 6], however, EF is not a perfect parameter for the diagnosis of cardiotoxicity, as it does not demonstrate early subtle changes and, when reduced, reflects a serious injury to the cardiomyocyte, followed by a poor outcome [7–9]. More recently, myocardial strain imaging has been shown to be more sensitive than LVEF in the characterization of LV systolic dysfunction following anthracyclines [10, 11]. Cardiac magnetic resonance (CMR) is considered the reference standard for the measurement of ventricular volumes and ejection fraction, mainly related to its advantages of providing unobstructed views of the heart in several planes and high reproducibility [12]. CMR also provides noninvasive assessment of myocardial tissue characteristics, by T1 mapping and derived extracellular volume fraction (ECV), T2 mapping and late gadolinium enhancement (LGE) imaging [13–15]. Cardiac magnetic resonance feature-tracking (CMR-FT) analysis offers a practical and rapid method to calculate strain from routinely acquired steady-state free precession (SSFP) cine images without the need of additional tagged sequences [16]. While changes in systolic function after anthracycline therapy have been described using CMR-FT [17–19], less data are available about late effects of chemotherapy in LV diastolic parameters, although diastolic dysfunction (DD) may precede systolic dysfunction [20–23], providing an earlier marker of cardiotoxicity. Therefore, in the present investigation, we sought to determine whether early diastolic strain rates (GDSR-E) by CMR-FT are impaired in long-term adult survivors of NHL. Our secondary objective was to analyze associations between GDSR-E with cumulative anthracycline dose, systolic function parameters and myocardial tissue characteristics.

## Methods

### Study design and participants

We conducted a cross-sectional observational study in a tertiary care center, with asymptomatic patients in remission of NHL who previously received anthracycline therapy and had finished their treatment at least 1 year before the enrollment. Patients were excluded if they had active cardiac disease, symptoms consistent with congestive HF, renal insufficiency or usual contraindications for CMR as implantable devices, cerebral aneurysm clips and cochlear implants. Subjects with no evidence of cardiovascular disease or neoplasia, recruited by open invitation, were included in the control group.

This study complied with the Declaration of Helsinki and was approved by the Research Ethics Committee of the Paulista Medical School—UNIFESP (approval number: 897.237) and Botucatu Medical School—UNESP (approval number: 969.316). All participants provided witnessed, written, informed consent.

### CMR technique and measurements

All participants underwent their examination on a 3.0-T Magnetom Verio Scanner (Siemens, Erlangen, Germany) with a phased array chest coil, according to study protocol. A cardiac cine steady-state free precession (SSFP) sequence was acquired using retrospective cardiac gating. Typically, 25 phases were acquired in 2-, 3-, and 4-chamber long axis views and a stack of short axis views. Scan parameters: field of view (FOV) 37-cm, repetition time (TR) 43.54 ms, echo time (TE) 1.38 ms, flip angle 50°, slice thickness 6 mm, in-plane image resolution 1.6 × 1.6 mm. Late gadolinium enhancement (LGE) with a single-shot phase-sensitive inversion recovery (PSIR) sequence was acquired in a stack of short axis, 2- and 4-chamber long axis views, 8 to 15 min after intravenous injection of 0.15 mmol/kg of a gadolinium-based contrast agent (gadoterate dimeglumine, Dotarem, Guerbet, France). Scan parameters: FOV 37-cm, TR 600 ms, TE 1.07 ms, flip angle 40°, slice thickness 5–8 mm, in-plane resolution 2.2 × 1.9 mm.

Quantitative T2 mapping was performed using a T2-prepared SSFP sequence with the following imaging parameters: FOV 36-cm, TR 254.32, TE 1.07 ms, flip angle 35°, slice thickness 8 mm, in-plane image resolution 2.5 × 1.9 mm, acquisition in late diastole on every fourth heartbeat; T2 preparations: 0 ms, 25 ms and 55 ms.

Quantitative T1 mapping was performed with a Modified Look-Locker Inversion-Recovery (MOLLI) sequence in mid-cavity short axis, pre-contrast (Native T1) and again at least 15 min after administration of gadolinium, according to previous published consensus [24]. Pre-contrast scan parameters: FOV 36-cm, TR 316.09, TE 1.12 ms, flip angle 35°, slice thickness 8 mm, in-plane image resolution 2.1 × 1.4 mm, acquisition in late diastole on every other heartbeat, minimal inversion time 120 ms; increment 80 ms. The T1 mapping scheme included 5 acquisitions after the first inversion pulse, followed by a 3-heartbeat pause and a second inversion pulse followed by 3 acquisitions [5(3)3]. Post-contrast scan parameters were the same, except by TR 396.09 and T1mapping scheme that included 4 acquisitions after the first inversion pulse, followed by a 1-heartbeat pause, a second inversion pulse followed by 3 acquisitions, 1-heartbeat pause and a third inversion pulse followed by 2 acquisitions [4(1)3(1)2].

### CMR analysis

The biventricular end-diastolic volume (EDV) and end-systolic volume (ESV) were measured by manual segmentation of the short axis cine images, using Argus function software (Siemens, Erlangen, Germany). The endocardial borders were traced at end-diastole and end-systole, including trabeculations and papillary muscles in the blood pool. EDV and ESV were calculated for each ventricle using the method of disc summation. Ventricular stroke volume (SV) was calculated with the difference between the EDV and ESV, and ventricular ejection fraction (%) was (SV/EDV) × 100. LV epicardial borders were draw only at end-diastole to calculate LV mass (M). All volume measurements were indexed for the body surface area (BSA) and expressed in ml/m^2^.

**Fig. 1:**
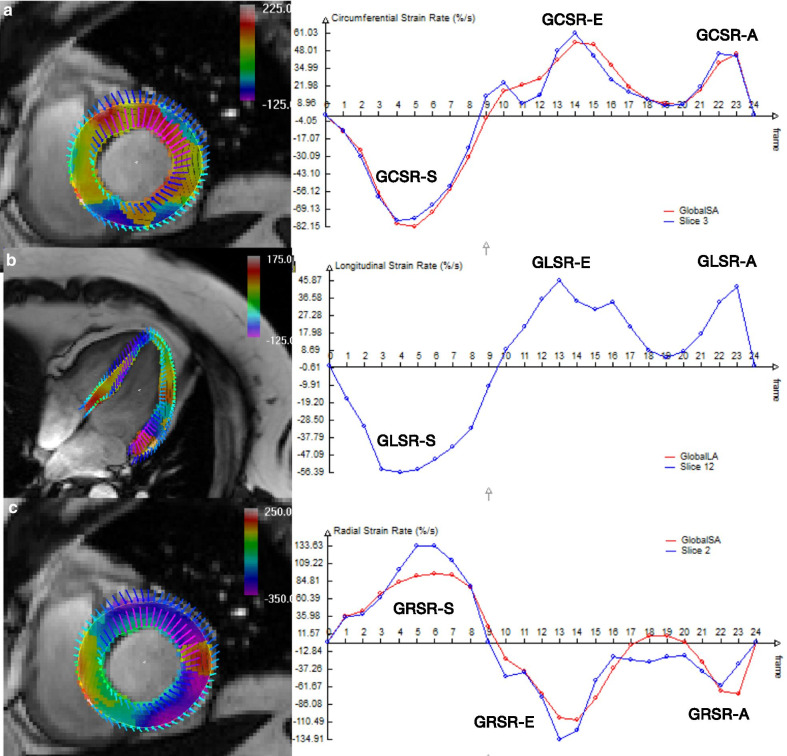
Representative strain rates profile from CMR-FT of a 64 years-old woman, treated with anthracycline therapy 10 years before CMR exam. This example demonstrates typical strain rates pattern with S (systolic), E (early diastolic), and A (late diastolic) waves in circumferential (**a**), longitudinal (**b**) and radial (**c**) directions

Strain analysis by feature-tracking was performed processing cine images with dedicated software (Trufi Strain, Siemens Healthcare, Medical Imaging Technologies, Princeton, NJ, USA) as previously described [18]. Circumferential and radial strains were analyzed in short axis stack by automatic segmentation of the LV blood pool cavity and myocardium, while longitudinal strains were obtained by manually tracing endocardial and epicardial contours in the first frame of 4-chamber long axis view and then automatically tracked to others frames. Strain values were obtained for each segment and global values defined as the mean of all segmental values. By convention, global longitudinal (GLS) and global circumferential (GCS) systolic strains are expressed as a negative value because it represents shortening of the myocardium relative to the original length, while global radial systolic strain (GRS) is expressed as a positive value because it represents thickening, with impaired GLS, GCS or GRS reflected by a value closer to zero. Otherwise strain rates reflects the velocities of deformation between frames. Diastolic strains are expressed as opposite of their counterpart systolic strains (i.e. GLS is expressed as a negative value and global longitudinal early diastolic strain rate (GLSR-E) as a positive value) (Fig. 1).

T1 and T2 maps were automatically generated on the MR scanner with motion corrected images using a novel non-rigid registration algorithm [25–27]. A region of interest (ROI) was then drawn conservatively in the septal myocardium for each map. In T1 maps another ROI was drawn in the blood pool to calculate ECV.

The ECV was calculated as (1-hematocrit) × (ΔR1_myocardium_/ΔR1_blood_), where Δ R1 = R1 post-contrast – R1 pre-contrast and R1 = 1/T1.

**Fig. 2:**
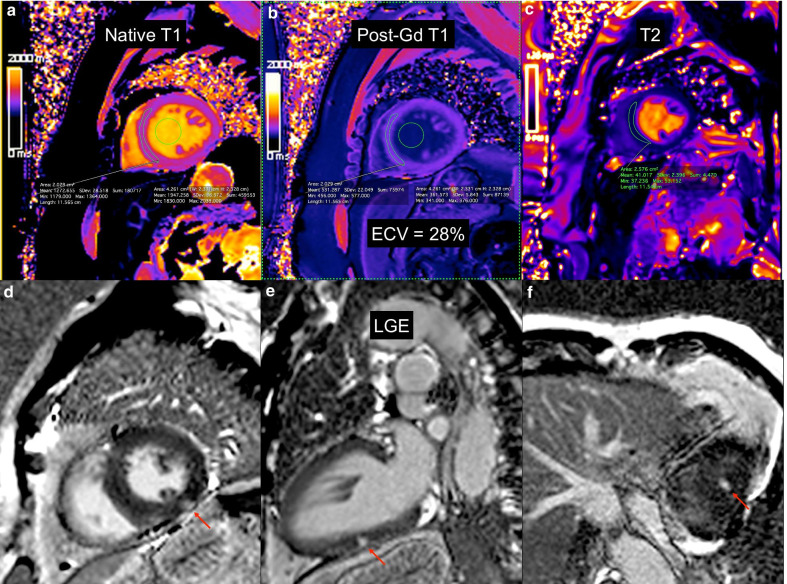
Example of tissue characterization by CMR. Native T1 (**a**), post-gadolinium T1 with derived ECV (**b**), T2 mapping (**c**) and LGE in short axis (**d**), 2-chamber (**e**) and 4-chamber (**f**) views. Red arrow points to focal non-ischemic LGE

Figure 2 shows an example of tissue characterization by CMR.

### Statistical analysis

Kolmogorov–Smirnov test was applied to determine appropriate parametric or nonparametric tests. Quantitative variables were expressed as mean ± standard deviation or median (interquartile range) and compared by Student's t test or Wilcoxon signed-rank test, whereas qualitative variables were expressed by their frequencies and percentages, and compared by the chi-square test or Fisher's exact test. Considering a mean difference in diastolic strain rates of 20 ± 20%/s, the alpha error of 0.05 and power of 0.8, for this study we calculated a number of 17 subjects per group. Spearman’s correlations were used to examine the relationship between continuous variables. Univariate linear regression analysis was used to evaluate the influence of clinical and CMR parameters in global circumferential early diastolic strain rate (GCSR-E), GLSR-E and global radial early diastolic strain rate (GRSR-E). Univariate variables with a significant correlation were entered into a stepwise multiple regression analysis to determine independent predictors of GCSR-E (model 1), GLSR-E (model 2) and GRSR-E (model 3). Data were analyzed using SAS Studio 3.8 and considered significant if *p* < 0.05.

## Results

### Clinical characteristics

We selected 20 adult survivors of non-Hodgkin lymphoma from the hospital records. One patient declined in participating and another one had claustrophobia during the CMR examination. So, the anthracycline group comprehended 18 adult survivors of non-Hodgkin lymphoma, treated with the same chemotherapy scheme (CHOP-cyclophosphamide, doxorubicin, vincristine, and prednisone), median cumulative anthracycline dose of 400 mg/m^2^ (IQR: 225–400 mg/m^2^), 14 (77.8%) males, between 32–81 years old (mean 57.6 ± 14.6 yo) that were studied 88.2 (± 52.1) months after exposure to therapy. Two (11.1%) patients received mediastinal radiotherapy at the time of treatment. The control group was composed of 17 subjects from the staff of the hospital or their relatives, without previous history of cardiovascular disease or neoplasia. There were no significant differences on clinical variables including cardiac risk factors between groups, except for age, marginally lower in controls (*p* = 0.047). The main clinical characteristics of the study population are shown in Table 1.

**Table 1:** Comparison of clinical variables between anthracycline and control groups

Variable	Anthracycline (n = 18)	Control (n = 17)	*p* value
Age (years)	57.6 ± 14.6	48.1 ± 12.2	0.047
Male (n, %)	14 (77)	8 (47)	0.601
Height (cm)	168.2 ± 10.2	164.1 ± 8.5	0.206
Weight (kg)	76.1 ± 15.3	69.7 ± 10.0	0.160
BMI (kg/m^2^)	26.8 ± 4.3	25.9 ± 2.9	0.469
BSA (m^2^)	1.86 ± 0.22	1.76 ± 0.15	0.157
Heart rate (bpm)	68.7 ± 14.3	66.2 ± 14.8	0.610
Blood hematocrit (%)	44.6 ± 3.7	44.2 ± 3.6	0.802
SBP (mmHg)	117.2 ± 13.4	120.5 ± 7.5	0.367
DBP (mmHg)	74.1 ± 8.4	76.9 ± 5.8	0.255
Smoking (n, %)	1 (5)	0 (0)	1.000
Hypertension (n, %)	5 (27)	4 (23)	1.000
Diabetes (n, %)	2 (11)	1 (5)	1.000
Dyslipidemia (n, %)	1 (5)	0 (0)	1.000
Framingham risk score (%)	7.8 ± 7.0	3.8 ± 6.6	0.099

Data expressed as mean ± standard deviation (SD) or n (%)

*BMI* body mass index, *BSA* body surface area, *SBP* systolic blood pressure, *DBP* diastolic blood pressures

### CMR parameters

**Fig. 3:**
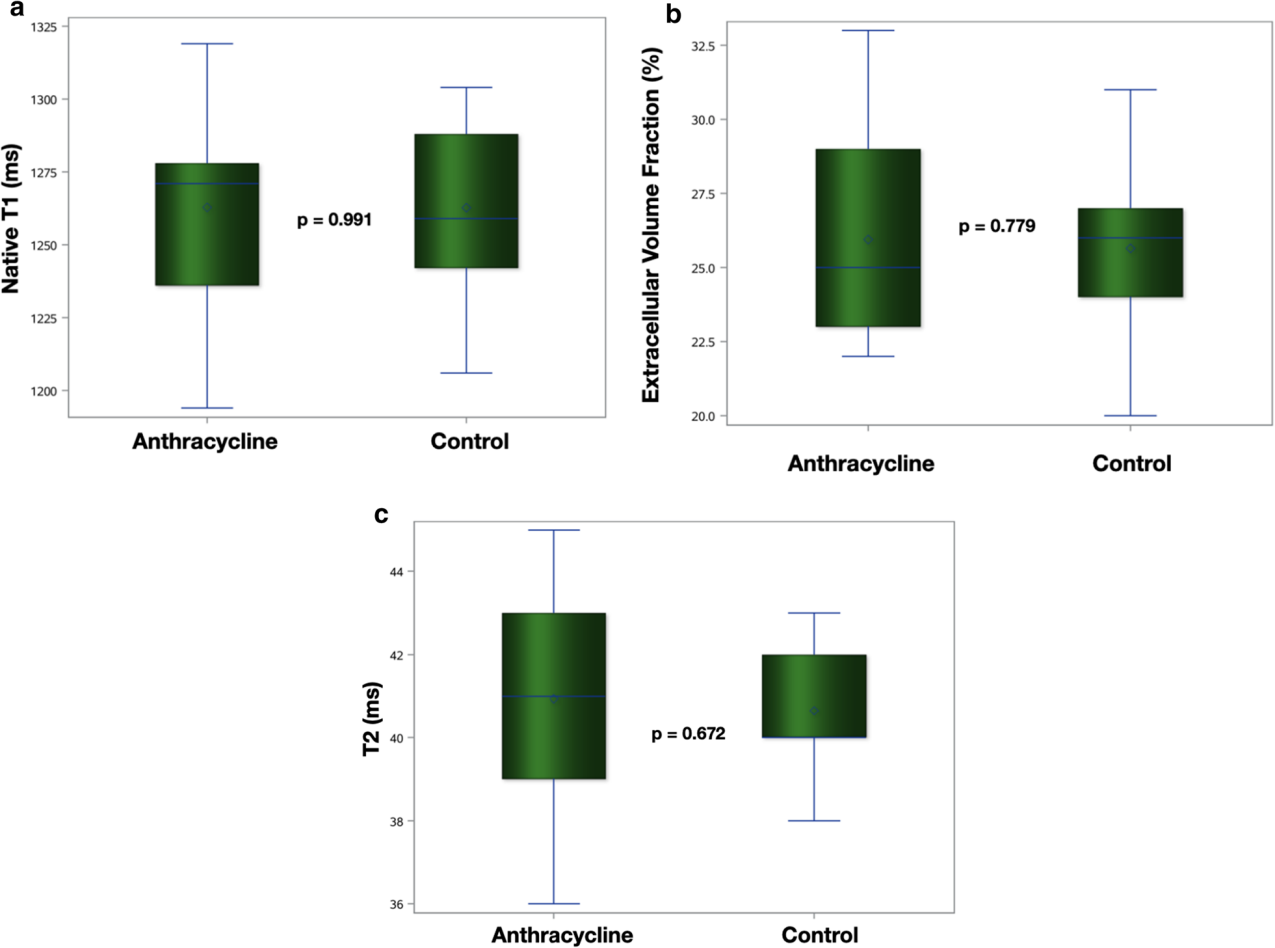
Box plots displaying median and range of values for myocardial native T1 (**a**), ECV (**b**) and T2 (**c**) in anthracycline and control groups

CMR measurements are summarized in Table 2. LV and right ventricular (RV) volumes, EF and LV mass were in the normal range, although LVEF was slight lower in anthracycline group (62.4% ± 7.5 vs 68.0% ± 4.6, *p* = 0.012), with a mild raise in LVESV (51.2 ml ± 16.3 vs 40.3 ml ± 14.9, *p* = 0.048). Focal, non-ischemic LGE was present in at least 01 myocardial segment in 03 patients (16.7%). Native T1, ECV and T2 were similar between anthracycline and control groups (Fig. 3). There were no significant differences in CMR parameters between the two patients who received mediastinal radiation and the other survivors in anthracycline group.

**Table 2:** Comparison of CMR variables between anthracycline and control groups

Variable	Anthracycline (n = 18)	Control (n = 17)	*p* value
LVEF (%)	62.4 ± 7.5	68.0 ± 4.6	0.012
LVEDV (ml)	135.8 ± 31.9	125.5 ± 38.3	0.395
LVESV (ml)	51.2 ± 16.3	40.3 ± 14.9	0.048
LVSV (ml)	84.5 ± 21.3	85.1 ± 25.4	0.932
LVM (g)	118.1 ± 23.6	111.6 ± 25.7	0.444
LVCM (g)	87.9 ± 20.4	83.2 ± 21.1	0.507
LVM/EDV (g/ml)	0.88 ± 0.13	0.92 ± 0.14	0.487
LVEDV index (ml/m^2^)	73.1 ± 16.1	70.2 ± 17.1	0.612
LVESV index (ml/m^2^)	27.5 ± 9.4	22.4 ± 7.1	0.085
LVSV index (ml/m^2^)	45.6 ± 10.7	47.8 ± 11.4	0.567
LVM index (g/m^2^)	63.7 ± 10.5	62.9 ± 9.9	0.823
LVCM index (g/m^2^)	47.5 ± 9.1	46.9 ± 8.5	0.841
RVEF (%)	63.7 ± 7.6	67.4 ± 6.8	0.143
RVEDV (ml)	132.3 ± 40.1	127.1 ± 40.5	0.701
RVESV (ml)	48.8 ± 19.8	42.9 ± 19.7	0.382
RVSV (ml)	83.4 ± 24.0	84.1 ± 23.3	0.933
RVEDV index (ml/m^2^)	70.7 ± 19.8	70.8 ± 18.3	0.994
RVESV index (ml/m^2^)	26.0 ± 10.5	23.4 ± 9.9	0.450
RVSV index (ml/m^2^)	44.6 ± 11.3	47.2 ± 10.2	0.479
Native T1 (ms)	1262.8 ± 35.5	1262.7 ± 31.4	0.991
ECV (%)	25.9 ± 0.35	25.6 ± 0.25	0.779
T2 (ms)	40.9 ± 2.4	40.6 ± 1.4	0.672

Data expressed as mean ± standard deviation (SD) or n (%)

*LVEF* left ventricular ejection fraction, *LVEDV* left ventricular end-diastolic volume, *LVESV* left ventricular end-systolic volume, *LVSV* left ventricular stroke volume, *LVM* left ventricular mass, *LVCM* left ventricular cardiomyocyte mass [(1-ECV × LVM], *RVEF* right ventricular ejection fraction, *RVEDV* right ventricular end-diastolic volume, *RVESV* right ventricular end-systolic volume, *RVSV* right ventricular stroke volume, *ECV* extracellular volume fraction

### Strain by CMR-FT

**Fig. 4:**
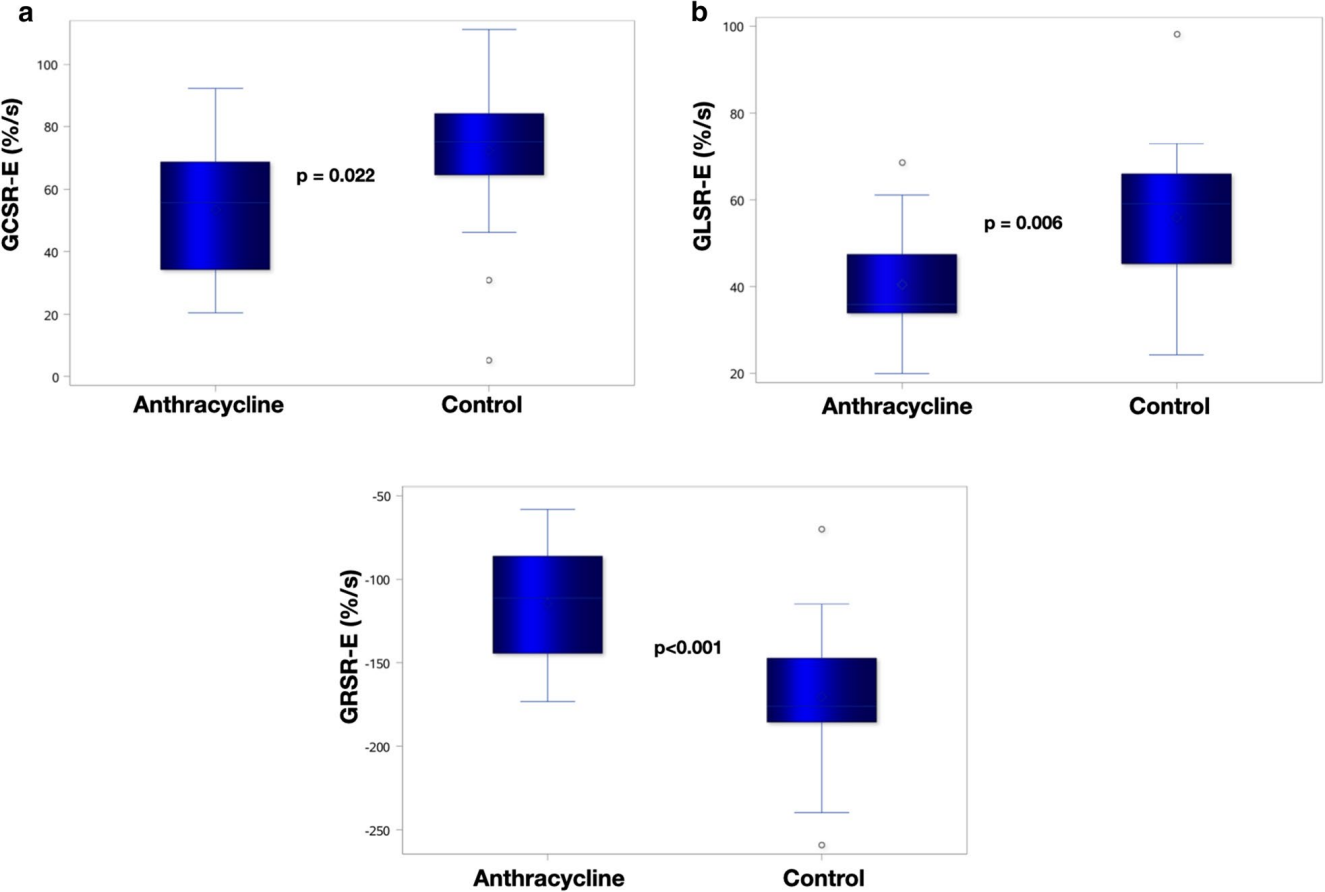
Box plots displaying median and range of values for GCSR-E (**a**), GLSR-E (**b**), GRSR-E (**c**) in anthracycline and control groups

**Fig. 5:**
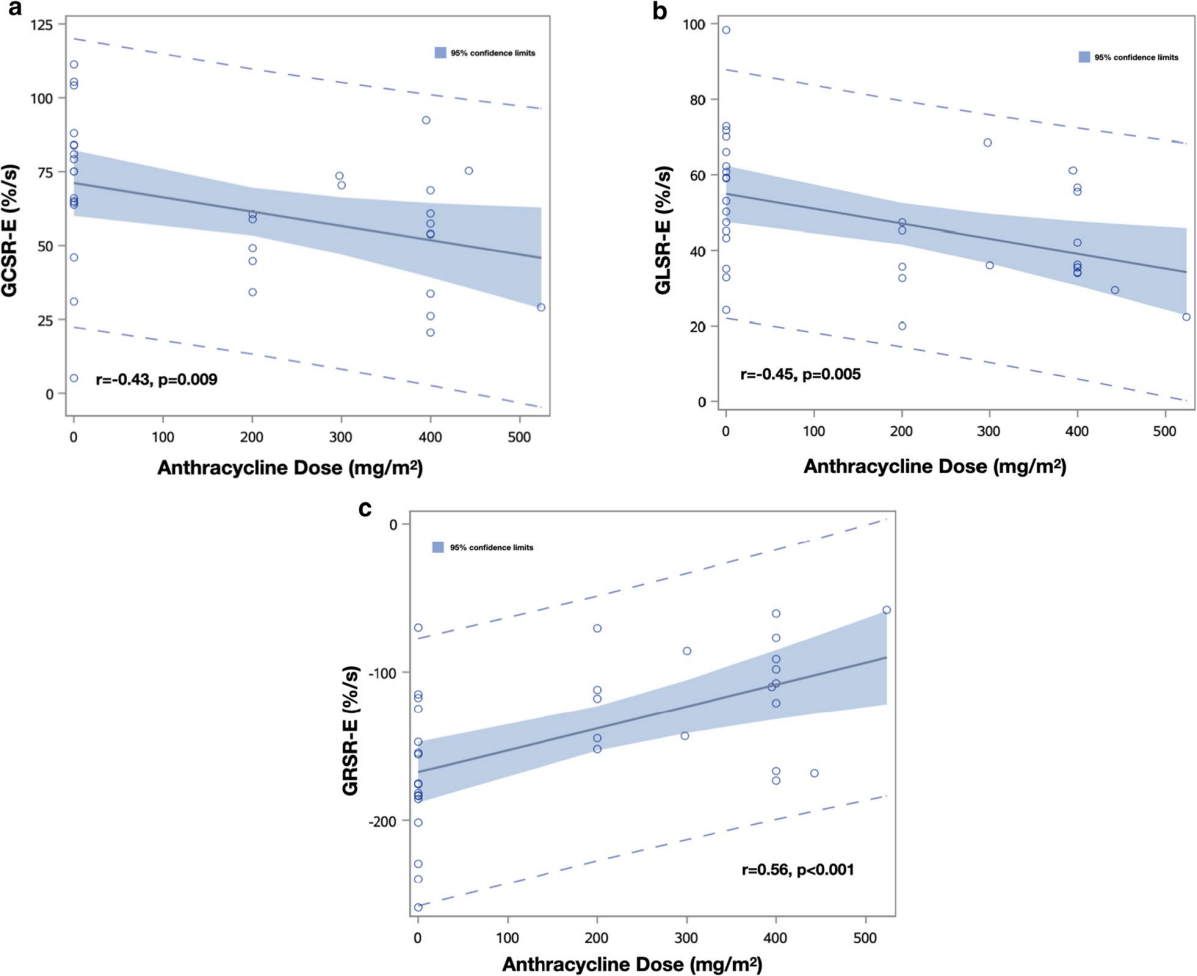
Relationship between GDSR-E and anthracycline dose. **a** A negative correlation was found between the GCSR-E and cumulative anthracycline dose (r = − 0.43, *p* = 0.009). **b** A negative correlation was found between the GLSR-E and cumulative anthracycline dose (r = − 0.45, *p* = 0.005). **c** A positive correlation was found between the GRSR-E and cumulative anthracycline dose (r = 0.56, *p* < 0.001)

**Fig. 6:**
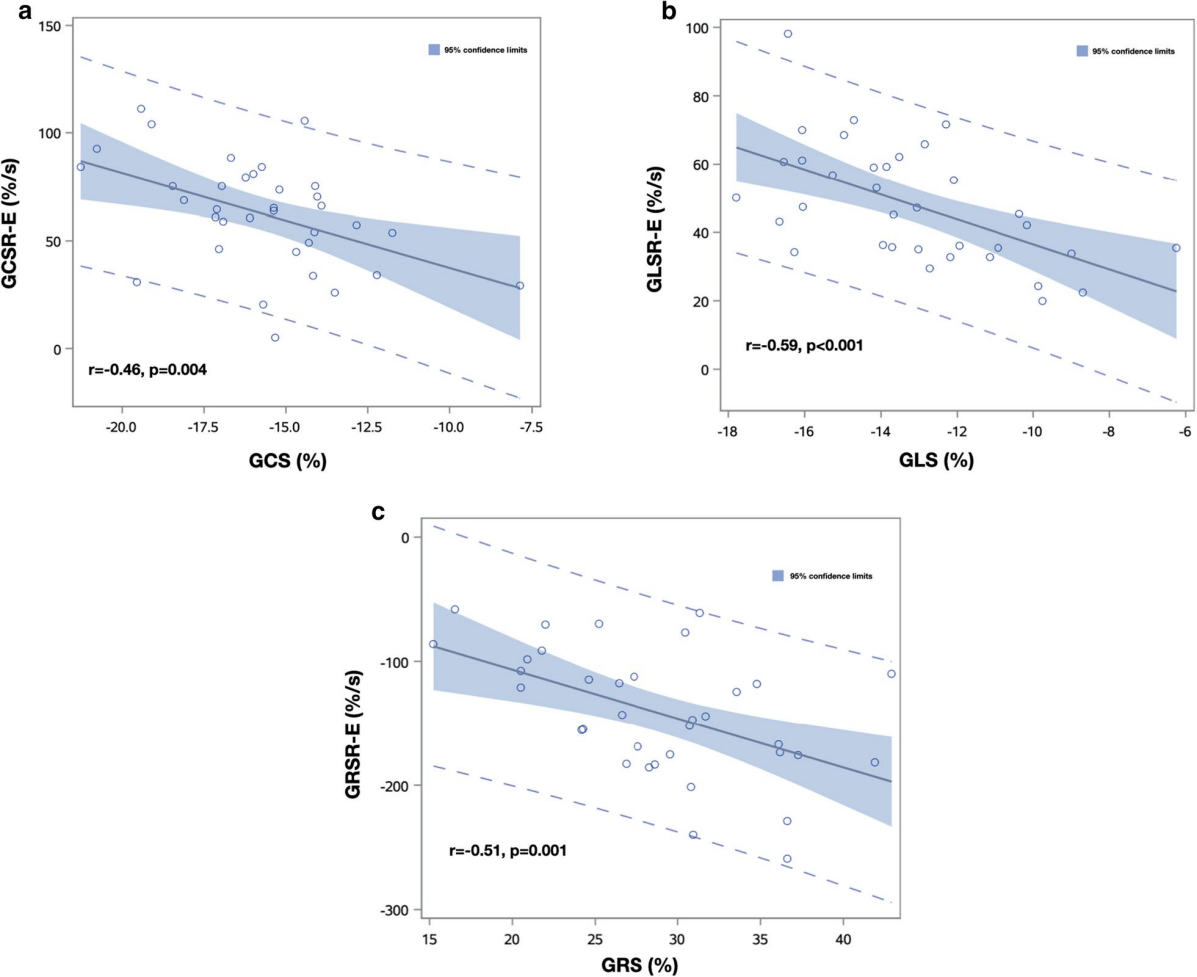
Relationship between GDSR-E and systolic strain. **a** A negative correlation was found between the GCSR-E and GCS (r = − 0.46, *p* = 0.004). **b** A negative correlation was found between the GLSR-E and GLS (r = − 0.59, *p* < 0.001). **c** A negative correlation was found between the GRSR-E and GRS (r = − 0.59, *p* < 0.001)

Strain parameters by CMR-FT are listed in Table 3. Compared with controls, anthracycline group showed impaired LV GCS, GLS, GCSR-E, GLSR-E and GRSR-E (Fig. 4). LV GCSR-E, GLSR-E and GRSR-E correlated with anthracycline dose (Fig. 5), LVEF and systolic strain in the same direction (Fig. 6). Univariate predictors of GCSR-E, GLSR-E and GRSR-E were cumulative anthracycline dose, age, LVEF and systolic strain. Systolic strain was an independent predictor of GCSR-E (Table 4), GLSR-E (Table 5) and GRSR-E (Table 6) on multivariate analysis, after correcting for age.

**Table 3:** Comparison of strain by CMR-FT between anthracycline and control groups

Variable	Anthracycline (n = 18)	Control (n = 17)	*p* value
LV-GCS (%)	− 14.8 ± 2.8	− 16.7 ± 2.1	0.026
LV-GCSR-S (%/s)	− 83.0 ± 18.0	− 88.3 ± 14.0	0.333
LV-GCSR-E (%/s)	53.5 ± 19.3	72.2 ± 26.7	0.022
LV-GCSR-A (%/s)	50.2 ± 19.2	54.4 ± 24.5	0.579
LV-GLS (%)	− 12.1 ± 2.9	− 14.1 ± 2.0	0.025
LV-GLSR-S (%/s)	− 61.2 ± 14.1	− 67.2 ± 11.4	0.179
LV-GLSR-E (%/s)	40.4 ± 13.0	55.9 ± 17.8	0.006
LV-GLSR-A (%/s)	41.9 ± 14.4	47.5 ± 15.7	0.282
LV-GRS (%)	27.3 ± 7.5	30.3 ± 5.2	0.184
LV-GRSR-S (%/s)	120.2 ± 31.4	129.8 ± 22.4	0.300
LV-GRSR-E (%/s)	− 114.4 ± 37.1	− 170.5 ± 48.0	< 0.001
LV-GRSR-A (%/s)	− 86.6 ± 51.0	− 80.3 ± 41.3	0.692
RV-GLS (%)	− 14.2 ± 3.7	− 15.4 ± 3.6	0.326
RV-GLSR-S (%/s)	− 70.5 ± 20.6	− 81.7 ± 23.9	0.147
RV-GLSR-E (%/s)	45.1 ± 20.1	56.0 ± 15.7	0.083
RV-GLSR-A (%/s)	59.9 ± 25.6	57.1 ± 24.5	0.736

Data expressed as mean ± standard deviation (SD)

*GCS* global circumferential strain, *GCSR-S* global circumferential systolic strain rate, *GCSR-E* global circumferential early diastolic strain rate, *GCSR-A* global circumferential late diastolic strain rate, *GLS* global longitudinal strain, *GLSR-S* global longitudinal systolic strain rate, *GLSR-E* global longitudinal early diastolic strain rate, *GLSR-A* global longitudinal late diastolic strain rate, *GRS* global radial strain, *GRSR-S* global radial systolic strain rate, *GRSR-E* global radial early diastolic strain rate, *GRSR-A* global radial late diastolic strain rate, *LV* left ventricular, *RV* right ventricular

**Table 4:** Determinants of global circumferential early diastolic strain rate

Independent variables	Dependent variable (GCSR-E)					
	Univariate analysis			Multiple analysis (r^2^ 0.44, *p* < 0.001)		
	B Coef	95% CI	*p*	B Coef	95% CI	*p*
Cumulative dose	− 0.04	(− 0.09;− 0.01)	**0**.**029**	–	–	–
Time after therapy	− 0.06	(− 0.25;0.13)	0.503	–	–	–
Age (years)	− 0.75	(− 1.30;0.19)	**0**.**009**	− 0.82	(− 1.29;− 0.35)	**0**.**001**
Gender (female)	6.09	(− 11.66;23.85)	0.489	–	–	–
Smoking (Yes)	− 18.38	(− 69.86;33.08)	0.472	–	–	–
Hypertension (no)	13.08	(− 6.14;32.31)	0.175	–	–	–
Diabetes (no)	− 16.32	(− 46.65;14.00)	0.281	–	–	–
Dyslipidemia (no)	1.79	(− 50.08;53.67)	0.944	–	–	–
Prior radiotherapy (no)	9.31	(− 27.78;46.40)	0.612	–	–	–
LVEF	1.64	(0.50;2.79)	**0**.**006**	–	–	–
LVMi	0.18	(− 0.67;1.05)	0.663	–	–	–
LGE (no)	8.99	(− 21.71;39.70)	0.555	–	–	–
Native T1	− 0.10	(− 0.36;0.15)	0.424	–	–	–
ECV	− 1.07	(− 3.89;1.75)	0.446	–	–	–
T2	1.56	(− 2.87;5.99)	0.478	–	–	–
GCS	− 4.37	(− 7.28;− 1.47)	**0**.**004**	− 4.74	(− 7.25;− 2.23)	** < 0**.**001**

*LVEF* left ventricular ejection fraction, *LVMi* left ventricular mass index, *LGE* late gadolinium enhancement, *ECV* extracellular volume fraction, *GCS* global circumferential strain

**Table 5:** Determinants of global longitudinal early diastolic strain rate

Independent variables	Dependent variable (GLSR-E)					
	Univariate analysis			Multiple Analysis (r^2^ 0.44, *p* < 0.001)		
	B Coef	95% CI	*p*	B Coef	95% CI	*p*
Cumulative dose	− 0.03	(− 0.06;− 0.01)	**0**.**009**	–	–	–
Time after therapy	0.07	(− 0.05;0.19)	0.245	–	–	–
Age (years)	− 0.28	(− 0.70;0.13)	0.173	− 0.43	(− 0.76;0.10)	**0**.**012**
Gender (female)	7.45	(− 4.70;19.60)	0.212	–	–	–
Smoking (Yes)	− 0.56	(− 36.63;35.51)	0.975	–	–	–
Hypertension (no)	9.12	(− 4.24;22.48)	0.174	–	–	–
Diabetes (no)	− 13.74	(− 34.65;7.16)	0.190	–	–	–
Dyslipidemia (no)	12.87	(− 22.91;48.65)	0.469	–	–	–
Prior radiotherapy (no)	− 2.80	(− 28.67;23.07)	0.827	–	–	–
LVEF	1.25	(0.47;2.02)	**0**.**002**	–	–	–
LVMi	− 0.25	(− 0.85;0.33)	0.384	–	–	–
LGE (no)	− 2.80	(− 28.67;23.07)	0.827	–	–	–
Native T1	− 0.05	(− 0.23;0.13)	0.559	–	–	–
ECV	0.33	(− 1.64;2.31)	0.730	–	–	–
T2	− 0.27	(− 3.41;2.87)	0.861	–	–	–
GLS	− 3.65	(− 5.53;− 1.77)	** < 0**.**001**	− 4.09	(− 5.86;− 2.33)	** < 0**.**001**

*LVEF* left ventricular ejection fraction, *LVMi* left ventricular mass index, *LGE* late gadolinium enhancement, *ECV* extracellular volume fraction, *GLS* global longitudinal strain

**Table 6:** Determinants of global radial early diastolic strain rate

Independent variables	Dependent variable (GRSR-E)					
	Univariate Analysis			Multiple analysis (r^2^ 0.64, *p* < 0.001)		
	B Coef	95% CI	p	B Coef	95% CI	p
Cumulative dose	0.14	(0.06;0.22)	**< 0**.**001**	–	–	–
Time after therapy	0.29	(− 0.04;0.64)	0.082	–	–	–
Age (years)	1.26	(0.07;2.45)	**0**.**037**	1.92	(0.99;2.85)	**< 0**.**001**
Gender (female)	− 21.70	(− 57.62;14.21)	0.227	–	–	–
Smoking (Yes)	24.36	(− 81.83;− 130.55)	0.643	–	–	–
Hypertension (no)	− 21.13	(− 61.05;18.78)	0.289	–	–	–
Diabetes (no)	0.85	(− 62.55;64.26)	0.978	–	–	–
Dyslipidemia (no)	32.56	(− 73.35;138.48)	0.535	–	–	–
Prior radiotherapy (no)	− 36.91	(− 112.26;38.43)	0.326	–	–	–
LVEF	1.64	(0.50;2.79)	**0**.**006**	–	–	–
LVMi	− 0.40	(− 2.18;1.36)	0.642	–	–	–
LGE (no)	− 49.56	(− 110.49;11.36)	0.107	–	–	–
Native T1	0.02	(− 0.51;0.57)	0.912	–	–	–
ECV	2.07	(− 3.73;7.88)	0.471	–	–	–
T2	− 3.87	(− 12.91;5.16)	0.389	–	–	–
GRS	− 3.94	(− 6.28;− 1.61)	**0**.**001**	− 5.05	(− 7.03;− 3.07)	**< 0**.**001**

*LVEF* left ventricular ejection fraction, *LVMi* left ventricular mass index, *LGE* late gadolinium enhancement, *ECV* extracellular volume fraction, *GRS* global radial strain

## Discussion

In the present investigation we demonstrated that GDSR-E measured by CMR-FT are impaired in adult survivors of NHL more than 7 years after anthracycline treatment. To the best of our knowledge, this is the first study focused in analyzing diastolic strain rates by CMR-FT in this setting. Moon et al. [28] found similar results using speckle-tracking echocardiography in children survivors of cancer. In their study, comparing with other strain parameters, diastolic strain rate showed the greatest percent difference following anthracycline exposure. The recent development of FT technology provided fast access to strain and strain rate measurements, in both systole and diastole, using SSFP cine images, without the need of additional tagged sequences acquisitions [29]. Measuring early diastolic strain rates, a surrogate for LV relaxation [30, 31], can improve the ability of CMR in detecting subclinical myocardial dysfunction in this population under high risk for heart failure, so our study addresses a clinically relevant question. Indeed, Serrano et al. [32] studying patients with breast cancer using TTE, at median follow-up of 12 months after anthracycline-based chemotherapy, found that none of the patients with normal diastolic function developed systolic dysfunction during follow-up.

In our study, impaired early LV relaxation, as assessed by GDSR-E, was associated with cumulative anthracycline dose suggesting that increased anthracycline dose is a risk factor for the development of DD. The dose-dependent anthracycline cardiotoxicity is a well-known phenomenon and early noninvasive imaging evidence of subclinical cardiovascular disease can occur even with low to moderate doses [10]. In fact, Rammeloo et al. [33] studying long-term childhood cancer survivors demonstrated that cumulative dose under 100 mg/m^2^ did not induce DD. The exact mechanism responsible for myocardial dysfunction as a result of anthracycline administration is unclear, however, the myocardial damage is thought to be mediated by intracellular oxidative stress that results in mitochondrial dysfunction, apoptosis, and myocyte necrosis. Damage to mitochondrial structure and function are one of the early cardiotoxic effects of doxorubicin [34, 35]. Early diastolic relaxation is an active energy-dependent process [36, 37], so it is expected that one of the earliest effects of anthracycline cardiotoxicity will be impaired relaxation. Different from us, studying trastuzumab-treated breast cancer patients, Gong et al. [38] found no consistent temporal changes in CMR-FT derived diastolic strain rate parameters after therapy and Reuvekamp et al. [39] demonstrated that an impairment of multigated radionuclide angiography (MUGA) derived diastolic parameters did not occur prior to systolic dysfunction. However, the mechanism of trastuzumab-related cardiotoxicity may be attributable to blockade of the HER2 receptor in cardiomyocytes, representing reversible type II cardiotoxicity without ultrastructural abnormalities, different from anthracycline dose-related type I cardiotoxicity [5].

The pathophysiology of diastolic dysfunction includes impairment of myocardial relaxation and stiffening of the myocardium. Alterations in myocardial collagen properties and an increase in ventricular fibrosis are known to result in reduced LV compliance [40, 41]. Histopathological early stages of anthracycline cardiomyopathy are characterized by myocardial edema, inflammation and vacuolization, whereas in the later stages, diffuse myocardial fibrosis predominates [42–44]. CMR has the unique ability to noninvasively characterize myocardial tissue, providing distinct biosignatures of early inflammatory involvement (raised native T1, ECV and T2) and late interstitial fibrosis and remodeling (raised native T1 and ECV but not T2) [45]. However, we failed to prove associations between GDSR-E and myocardial tissue characteristics. Our study may have been underpowered to demonstrate this association as it was not our primary objective, although stiffening of the myocardium by edema or fibrosis could be associated with a more restrictive filling pattern rather than impaired relaxation, suggesting GDSR-E could conceptually be a more sensitive and earlier marker of cardiotoxicity.

We found a significant association between GDSR-E and systolic function parameters (LVEF and systolic strain), and in a multivariate analysis reduced systolic strain was an independent predictor of impaired early diastolic strain rates, even when age was considered as confounder. Similar to us, Stoodley et al. [46] reported altered diastolic strain by TTE and its association with systolic dysfunction 1 week after anthracycline therapy and Boyd et al. [47] showed that anthracycline-related diastolic dysfunction was more common in the subgroup with reduced GLS compared to those without changes in GLS. These findings could be explained because GDSR-E provide similar diagnostic information as LV regional lengthening velocity (E’) measured noninvasively by tissue Doppler imaging and reflects relaxation as well as restoring forces [48]. Restoring forces are generated in systole by a complex set of mechanisms, LV myocardial wall stores energy in the form of elastic recoil, and this energy is released when the myocardium relaxes. So, GDSR-E are determined in part by systolic function, reflecting the tight coupling between systolic and diastolic function [49]. Indeed, in a recent study Ito et al. [50] demonstrated that among heart failure with preserved ejection fraction patients, which anthracycline related cardiotoxicity could represent a subset, CMR-FT GLS was independently associated with invasive measures of LV relaxation.

### Study limitations

This study is limited by the small sample size and cross-sectional design, so further corroboration of these findings in prospective larger-scale multicenter studies is required. Likewise, we cannot say whether the impairment in GDSR-E occurred previously or concomitantly with the reduction in systolic strain. As anthracycline patients were marginally older than controls we cannot exclude some influence of the age in the observed difference of GDSR-E measures between the two groups. Also, longer follow-up is necessary to determine if GDSR-E measured by CMR-FT will predict the development of symptomatic heart failure or will be helpful in guiding therapy. We studied only long-term survivors instead of all patients treated with anthracycline-based chemotherapy in our institution, therefore some bias of selection has to be considered.

## Conclusions

In this study we demonstrated that left ventricular GDSR-E by CMR-FT are impaired late after anthracycline chemotherapy in adult survivors of NHL. Impaired GDSR-E were associated with cumulative anthracycline dose and systolic function parameters. There were no correlations between GDSR-E and myocardial tissue characteristics.

## Data Availability

The datasets used and/or analyzed during the current study are available from the corresponding author on reasonable request.

## Abbreviations

BSA: Body surface area
CMR: Cardiac magnetic resonance
CMR-FT: Cardiac magnetic resonance feature-tracking
DD: Diastolic dysfunction
ECV: Extracellular volume fraction
EDV: End-diastolic volume
EF: Ejection fraction
ESV: End-systolic volume
FOV: Field of view
GCS: Global peak systolic circumferential strain
GCSR-A: Global circumferential late diastolic strain rate
GCSR-E: Global circumferential early diastolic strain rate
GCSR-S: Global circumferential systolic strain rate
GDSR-E: Early diastolic strain rates
GLS: Global peak systolic longitudinal strain
GLSR-A: Global longitudinal late diastolic strain rate
GLSR-E: Global longitudinal early diastolic strain rate
GLSR-S: Global longitudinal systolic strain rate
GRS: Global peak systolic radial strain
GRSR-A: Global radial late diastolic strain rate
GRSR-E: Global radial early diastolic strain rate
GRSR-S: Global radial systolic strain rate
HF: Heart failure
LGE: Late gadolinium enhancement
LV: Left ventricle/ventricular
MOLLI: Modified Look-Locker inversion-recovery
NHL: Non-Hodgkin lymphoma
PSIR: Phase-sensitive inversion recovery
ROI: Region of interest
RV: Right ventricle/ventricular
SD: Standard deviation
SSFP: Steady-state free precession
SV: Stroke volume
TE: Echo time
TR: Repetition time
TTE: 2-Dimensional transthoracic echocardiography
